# Churches and Charity Funds

**Published:** 1917-01-20

**Authors:** J. Allen Bell

**Affiliations:** Vicar of Wimbledon. The Vicarage, Wimbledon, S.W.


### THE EDITOR'S LETTER-BOX.

### Churches and Charity Funds.

*To the Editor of* THE HOSPITAL.

SIR,—As you have been good enough to send me a copy of your paper dated January 6 and to draw my attention to the article on "Immoralities in Church Management," perhaps you will allow me to write some few words in reply.

First let me say that I am able to do this without bias, as it is contrary to our practice in this parish to make such deductions. But as I have had many years' experience of church finance, I know how much there is to be said for the practice you condemn. Take the recent Red Cross Sunday for an example. To my personal knowledge, the churchwardens of three separate churches made representations to their clergy as to the unfairnesss of taking from them the offerings of a *second* Sunday in 1916 for Red Cross work. They had heavy expenses to meet at the end of the Christmas quarter, and the last Sunday in the year had been relied upon to bring them in a considerable amount.

I can well conceive that in some similar case a vicar might feel the strength of his churchwardens' case, and might quite properly reply, "We ought not to stand out of a national movement, but we must not be generous at the expense of our creditors, therefore we will give notice to the congregation that the first charge upon the collection will be the amount we should naturally collect —an average collection—for church expenses, and the balance will be given to the Red Cross Fund." No regular worshipper would be likely to object to a procedure which he would recognise as straightforward and just. Possibly a stranger, anxious to use the service as a means of contributing to the special fund, might regret that all his money could not go as he would desire, but a little thought might convince him that even he could have no real right to complain. For what is his position? He uses a particular church as an instrument of his benefaction. He finds the church warmed, clean, and properly appointed, and he must know that this result cannot be reached without cost.

Even in hospitals the contribution that an individual donor might choose to send is not fully available for the purpose the donor has at heart. A percentage of it is expended in the prosaic but necessary costs of collection, office expenses, administrative expenditure, and the like. Would not churchwardens who know their own difficulties be acting on the same lines if they should choose to say to the Red Cross Committee, "We will give you the opportunity of our well-attended services, we will provide all the accessories, we will help our clergy to press upon the congregation the importance of the occasion, but we cannot consent to rob St. Peter to pay St. Paul, and therefore you must not ask us to forgo that proportion of the offerings which otherwise we should receive, and which is vital to our needs"?

Every year the problem of church finance becomes more serious, as the number of societies claiming church collections grows. If the principle enunciated in your paper were accepted, the alternative would be for churchwardens to refuse all societies the unique opportunity which church services provide. Immorality in the matter could only be charged if any part of the collection were kept back, when, without any reservation, the notice had been given that the alms would be for some special fund. But that, I think, is a course which neither clergy nor churchwardens would be likely to countenance.—Your obedient servant,

J. ALLEN BELL,
Vicar of Wimbledon.

The Vicarage, Wimbledon, S.W.
January 13, 1917.

*** It is a question of system. The stranger would and does give to expenses when opportunity offers. We have dealt with most of the points in this letter in an article on page 312. It is clear that the expenses of maintaining God's House must be provided by the congregation of every unendowed place of worship, and this should be readily done when the management is businesslike and the ministrations are worthy and sincere. What is wanted is uniformity in method, and the abolition of existing abuses of loose methods in some places of worship. These reforms could readily be secured by the adoption of some such plans as those recommended in the article referred to. Will not the Council of the Metropolitan Hospital Sunday Fund and of other Sunday Funds throughout the country use their great influence to secure the desired result?—ED. THE HOSPITAL.

